# Charting a new course: Recommendations for strategic priorities for the African Medicines Agency

**DOI:** 10.4102/jphia.v17i1.1712

**Published:** 2026-06-20

**Authors:** Olawale Salami, Emmanuel Agogo

**Affiliations:** 1 Clinical Trials Unit, Institute of Global Health Equity Research, University of Global Health Equity, Kigali, Rwanda; 2 Department of Pandemic Preparedness, FIND Diagnostics, Geneva, Switzerland

**Keywords:** African Medicines Agency, regulatory harmonisation, clinical trials, drug development, regulatory authority, access to medicines

## Abstract

This article examines the strategic landscape facing the inaugural leadership of the African Medicines Agency. It outlines the core challenges constraining Africa’s pharmaceutical sector: fragmented regulatory frameworks, inadequate clinical trial regulation, limited capacity for drug development (notably for neglected and emerging diseases) and slow access to novel medicines. Drawing upon the history of regulatory harmonisation in Africa, international best practices, and current continental initiatives, the article proposes a comprehensive agenda for leadership and operational priorities designed to strengthen regulatory performance, support domestic manufacturing, ensure ethical and efficient trial oversight, and broaden access to innovation. The conclusion calls for multisectoral collaboration, innovative financing and sustained political commitment to realise the vision of Agenda 2063 and universal access to quality medical products in Africa.

## Introduction

Africa remains disproportionately burdened by infectious diseases, non-communicable illnesses and maternal and childhood mortality^1^; yet, it contributes only a fraction of global clinical trials^2^ and pharmaceutical production.^3^ Regulatory delays and a lack of harmonisation further exacerbate medicine inaccessibility and perpetuate reliance on health aid for low-income countries.^4^ The establishment of the African Medicines Agency (AMA) is recognised as a cornerstone of the African Union (AU) Agenda 2063 and the Pharmaceutical Manufacturing Plan for Africa,^5^ with the mandate to enhance African self-reliance in health while aligning with continental free trade ambitions.^6^

The appointment of the inaugural Director General of the AMA marks a pivotal moment in Africa’s health governance.^7^ As former Chief Executive of the Ghana Food and Drugs Authority and Chair of the African Vaccines Regulatory Forum, the new Director General brings extensive regulatory and clinical trial oversight expertise. In addition, strong experienced leadership at the AMA is critical to achieve continental goals of regional and local manufacturing. Among the most significant bottlenecks to the rollout of medical countermeasures (diagnostics, vaccines and other health commodities) during the coronavirus disease 2019 (COVID-19) response in Africa was the chaotic and duplicative regulatory systems on the continent.^8^ Therefore, there is a need for institutional mechanisms to ensure that these products are of the requisite quality and standards.

This article systematically analyses the continental imperatives faced by regulatory authorities across the continent. It articulates strategic priorities to address drug development bottlenecks, regulatory fragmentation, ethical and operational weaknesses in clinical trial supervision and patchy access to innovative medicines. We focus on four key themes: strategic context, operational priorities, implementation pathways and concluding reflections. The core argument is that the new leadership can achieve a harmonised, capacity-enhancing, ethically robust, innovation-friendly and access-oriented regulatory architecture.

## Strategic context

Since the 2009 establishment of the African Medicines Regulatory Harmonisation (AMRH) initiative, efforts have been made to align regulatory practices across Regional Economic Communities (RECs), yet progress has been uneven.^5^ On the African continent, only nine countries (Egypt, Ghana, Nigeria, South Africa, Tanzania, Zimbabwe, Rwanda, Senegal and Ethiopia) are classified by the World Health Organization (WHO) as having regulatory systems of maturity level 3 as of October 2025, and no African country is considered a Statutory Regulatory Authority (SRA) or a WHO Listed Authority.^9^ Clinical trials continue to be concentrated in a few countries, notably South Africa and Egypt, which undermines equity in research participation and capacity development.^10^ The lack of a coordinated mechanism perpetuates the reliance on parallel multiple approval systems across national boundaries, which impedes rapid access to life-saving innovations, especially during health emergencies such as cross-border outbreaks, epidemics and pandemics.^11^

The AMA Treaty, adopted in February 2019 and entered into force on 05 November 2021 after ratification by 15 Member States, frames the agency’s remit, encompassing medicines, vaccines, medical devices, diagnostics and traditional therapies.^6^ It aims to harmonise legislation, coordinate joint lists of essential medicines, accredit laboratories, execute joint dossier reviews and inspect manufacturing facilities. The vision is guided by AU Agenda 2063, seeking unity, health security, local pharma production and universal health coverage.^12^ With headquarters in Rwanda and an inclusive Governing Board, the AMA is positioned to transcend regional silos, foster a new era of regulatory harmonisation on the African Continent and fast-track access to new technologies and health products.

The need to streamline the transition of roles and handover of responsibilities to AMA is also an important consideration. The AMRH programme was started in 2009 as a response to addressing challenges faced by National Medicine Regulatory Authorities (NMRAs) in Africa. This initiative is housed by the African Union Development Agency – New Partnership for Africa’s Development (AUDA–NEPAD) in collaboration with the Africa Centres for Disease Control and Prevention (CDC), which will need to step back and transition all interventions to the new agency.

## Operational priorities

As the nascent agency commences operation, the following priorities should be considered by the new leadership of the AMA.

### Advocacy for more countries to ratify the treaty

The agency will have to be the leadership’s most ardent advocate by ensuring that other African countries buy into the initiative and ratify the treaty. This is essential for establishing credibility and further solidifying the mandate of the organisation on the continent. Currently, only 29 countries have completed the ratification and deposited their instruments^13^ (Table 1). Furthermore, the AMA should advocate for sustainable financing models, including cost recovery, donor support and Member State contributions, ensuring independence and long-term viability of the agency.

**TABLE 1:** Ratification status of the African Medicines Agency treaty among African Union member states (reference).

Category	Number	Details
Total AU Member States	55	All AU countries.
Ratified the AMA Treaty	31	Countries that have completed ratification and deposited instruments.
Initial Ratifiers (2021)	17	Algeria, Benin, Burkina Faso, Cameroon, Chad, Gabon, Ghana, Guinea, Mali, Mauritius, Namibia, Niger, Rwanda, Seychelles, Sierra Leone, Tunisia and Zimbabwe.

AU, African Union; AMA, Africa Medicines Agency.

### Harmonisation of regulatory frameworks

The primary task of the Directorate is to deepen regional and continental harmonisation. This will involve finalising and promoting the adoption of the AU Model Law on Medical Products Regulation across Member States, a critical step to ensure regulatory coherence.^12^ Building upon AMRH structures,^13^ the AMA must set up processes to fast-track the systems to standardise dossier requirements, evaluation pathways and quality management systems, leveraging digital platforms and interoperability to reduce duplication and accelerate reviews.^14^ Lessons learnt, best practices in regulatory agility and experiences from countries such as Ghana, South Africa and other Member States with good regulatory performance should be leveraged and adopted as routine practice.

Aligning pathways for biosimilars and complex therapies demands investment in regulatory science, training and expert networks. Collaboration with the European Medicines Agency, the WHO-listed regulators and other agencies can facilitate regulatory reliance models and harmonised joint dossier assessments. Through such initiatives, the AMA can reduce approval timelines, increase transparency and contribute to continental trade integration under the African Continental Free Trade Area (AfCFTA).

### Strengthening national and regional capacities

Africa’s regulatory landscape exhibits significant variation in infrastructure, personnel and resources. Strengthening institutional resilience is therefore a top priority. African Medicines Agency’s mandate should include accreditation and oversight of Regional Centres of Regulatory Excellence (RCORE), where regulatory training, Good Laboratory Practice (GLP)-certified laboratories, pharmacovigilance systems and Good Manufacturing Practices (GMP) inspection will be co-ordinated. A strategic workforce development agenda encompassing training in regulatory science, translational research, legal aspects and technical competency (e.g., dossier assessment, clinical trial oversight) is critical.

Academia and professional bodies must be engaged in establishing curriculum standards for improved regulatory expertise and competencies. Postgraduate regulatory science programmes collaborating across universities will build Africa’s long-term capacity.

### Ethical and efficient clinical trial oversight

Robust clinical trial oversight lies at the intersection of science, ethics and public trust. Competition between national approval authorities and inconsistent standards has discouraged international research sponsorship.^15^ Under the new stewardship, the AMA must launch a centralised clinical trial review system with single-entry portals, mutual recognition and standardised ethics committee procedures.

Harmonised clinical trial regulations will attract sponsors, facilitate local innovation and reduce trial costs.^16^ The AMA should also strengthen capacity for trial monitoring, biostatistics, data safety monitoring boards and digital case report forms, thereby assuring integrity. Anti-counterfeit surveillance and pharmacovigilance networks complement these efforts and safeguard post-approval safety, both critical components given the prevalence of substandard medicines.

It is essential that ethical research practices and conduct be promoted. Strict penalties and transparency will lead to the avoidance of unethical research and corruption in clinical trials.^17^

### Fostering innovation and local production

The AMA has a mandate to catalyse industrial-scale pharmaceutical manufacturing across the continent. To this end, it should collaborate with national regulatory bodies and manufacturers to enable technology transfer, expedite local approval of WHO-prequalified products and establish manufacturing partnerships across RECs.

Regulatory flexibility to support regional drug production includes accepting clinical data from comparable jurisdictions, fast-track approvals for innovations addressing neglected or epidemic diseases and supply chain resilience protocols.

Systems for supporting new in-class technological platforms, diagnostic innovations and digital innovations are essential. Promotion of culturally relevant health products and consideration of excipients that may have an impact on the use and acceptance of products need to be catalogued and understood. Public–private dialogues, incentives and GMP certification of African manufacturing facilities by the AMA will enhance confidence, reduce imports and drive economies of scale.

### Equitable access to innovative medicines and health products

Africa’s access to novel treatments, such as Messenger ribonucleic acid (mRNA) vaccines, oncology therapies and advanced biologics, is limited by regulatory roadblocks, high prices, and supply challenges.^18^ African Medicines Agency must assume a proactive role in defining continental access strategies by examining the reliance on WHO prequalification, which has been seen as a barrier to access, issuing early scientific advice and supporting rolling submissions for essential innovations.

The role of AMA in negotiating pooled procurement should be considered very closely and guided by transparency, and with the best interests of the continent at the centre of any such proposals.

Coordination with donor agencies, Gavi, Coalition for Epidemic Preparedness Innovations (CEPI) and continental health financing instruments can enable tiered pricing and licence agreements appropriate to African markets. The expertise and experience of the new leadership with Ghana’s Food and Drugs Authority and global research and development advisory boards provide a unique opportunity to lead on acceleration schemes for product evaluation and approval in Africa.

## Implementation pathways

In order to move this aspiration forward, we have identified good governance, digital platforms, political will and transparency as critical enablers that underpin all operational priorities.

Firstly, the AMA’s governance must balance AU representation, including Africa CDC and AUDA-NEPAD, regulatory independence, technical competence and stakeholder inclusivity. The Governing Board should incorporate RECs, Medicine Regulatory Authorities (MRAs), industry groups, civil society and academic leaders. An efficient Secretariat that routinely reports clear performance indicators and regional representation offices will boost effectiveness and accountability. Systems for the separation of powers and effective oversight of the executive and technical functions will build trust and accountability. In addition, the transition of AMRH activities to the AMA should be clearly articulated, and we call for close collaboration with AU agencies to lead or co-lead specific activities, fostering a spirit of partnership while minimising duplication and overlap.^19^

Secondly, the deployment of interoperable, cloud-based, digital platforms for dossier submission, pharmacovigilance, GMP tracking and joint clinical trial reviews can dramatically increase efficiency and reduce administrative overhead. Existing systems such as the Afrivigilance^20^ will need to be strengthened. The AMA should invest in cybersecurity and ensure that all regulatory information systems, data analytics for safety monitoring, and interoperable databases cannot be tampered with or hacked. Digital innovation from the outset will also support transparency, enabling real-time tracking and reporting, bolstering public trust and enabling evidence-based policymaking.

Raising political will among AU Member States remains critical. The AMA should articulate clear and critical value propositions, such as time-savings, cost-effectiveness and public health impact to justify sustained financing for its activities. Blended funding models combining Member State fees, donor grants and revenue from services should be explored. Demonstrating early successes, such as reduced approval times, quality improvements or expanded trial activity, will catalyse further support.

The AMA is a gateway to the health of citizens and the health economies of the countries of Africa. This makes it doubly important to ensure that the interests of the Member States are central to the work that the agency does. Avoiding donor dependence or a situation where the agency’s major funders have vested interests will ensure that the agency’s outcomes can be independent and trustworthy. Clear and frequent communication via well-established channels has to be the norm. This is critical to avoiding misinformation and disinformation, which in the long run can damage the credibility and acceptance of the guidance from the AMA.

## Conclusion

The COVID-19 pandemic and emerging epidemics have underscored the urgency of continental self-reliance in health. The AMA has the potential to prevent future supply disruptions, reduce drug costs and stimulate Africa-led research. By embedding ethical, scientific, and innovation-driven values, the AMA can transform health outcomes and elevate Africa’s standing in global pharmaceutical development. Success will align Africa with Agenda 2063 goals, drive universal health coverage and inspire confidence in the continent’s capability to oversee its pharmaceutical destiny. The success of AMA hinges on delivering strategic wins across regulatory harmonisation, capacity development, trial oversight and equitable access.
